# Performance evaluation and optimization of a *Moringa Oleifera* depodding machine: A response surface approach

**DOI:** 10.1016/j.heliyon.2020.e03465

**Published:** 2020-02-25

**Authors:** Clement Adekunle Komolafe, Peter Pelumi Ikubanni, Clinton Emeka Okonkwo, Faith Olusola Ajao, Adewumi Samuel Alake, Tajudeen M. Adeniyi Olayanju

**Affiliations:** a Department of Mechanical Engineering, College of Engineering, Landmark University, P.M.B. 1001, Omu Aran, Nigeria; b Department of Agricultural and Biosystems Engineering, College of Engineering, Landmark University, P.M.B. 1001, Omu Aran, Nigeria

**Keywords:** Agriculture, Industrial engineering, Mechanical engineering, Performance efficiency, Moringa, Depodding, Response surface analysis, Speed of rotation, Moisture content

## Abstract

Depodding of moringa which is still being carried out manually by removing with hand or by hitting a bag containing the pods is time-consuming, labour intensive and not economical. The demand for quality oil-bearing moringa seeds that have a wide area of industrial applications necessitates innovative deppoding techniques that will improve its market value. To ameliorate these problems, moringa depoddding machine has been developed but studies on performance evaluation and optimal parameter setting are sparsely reported. This study therefore, evaluated the effects of the processing factors (moisture content (MC) and speed of rotation (SR)) levels on the performance (throughput capacity (TP), effective throughput capacity (ETP), labour requirement (LR), depodding coefficient (DC), coefficient of wholeness (CW), depodding efficiency (DE), depodded kernel (DK), undepodded kernel (UK), small broken kernel (SBK), and big broken kernel (BBK)) of the designed and fabricated moringa depodding machine using the response surface methodology and test between subjects-effects. The experimental design used was a two factor, three levels i-optimal randomized design. Mathematical models relating the process factors to performance were developed. The predicted optimum results obtained were validated using the observed values of the experiment. MC and SR were found to have a significant effect on the performance of the machine. The predicted optimum performance of the machine were 113.73 kg/hr, 109.45 kg/hr, 0.85 man-hour required/Kg, 96.15 %, 0.96, 93.93 %, 0.98, 0.02, 10.64 %, and 1.24 % for TP, ETP, LR, DC, CW, DE, DK, UK, SBK, and BBK respectively at MC and SR of 10.10 % wet basis and 564 rpm. The experimental values at these processing conditions were close to the predicted optimum results obtained with little deviations which were statistically insignificant. The selected models sufficiently predicted the performance of the developed machine.

## 1 Introduction

**Figure 1:**
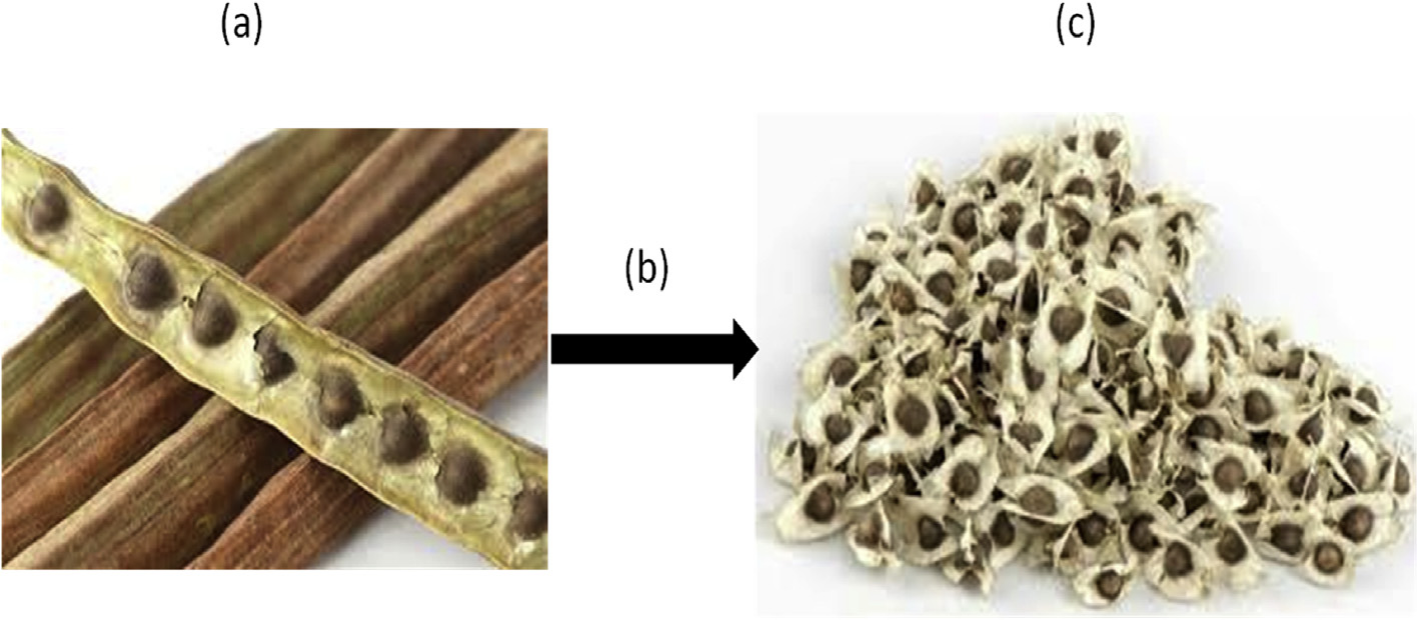
(a) moringa pod; (b) depodding operation; (c) undehulled moringa seeds.

*Moringa oleifera* plant is rich in protein and bioactive compounds like essential oils, saponins, and tannins with several industrial uses [1, 2, 3]. The tree produces fruits in pod form having drumstick shape which houses the undehulled seeds as in Figure 1 [4].

The process of depodding is a size reduction activity of breaking the case containing the seeds [5]. Depoddingof moringa fruit is the first basic unit operation that must be carried out before other post-harvest processes such as dehulling/shelling, cleaning, and oil expelling depending on its end-use. The depodding process of moringa is still being carried out manually, by hitting a bag containing moringa pods with a wooden stick or removing them by hand. This manual method is time-consuming, causes high mechanical damage to the product, with a lot of drudgeries attached to its process. Falade and Aremu [3] stated that manual processing of moringa seed is expensive, thereby damping its economic viability.

Depodding machine for various crops has been reported (Iyanda *et al.* [5], Adewumi and Fatusin [6]) for cocoa; ([Oloko and Agbetoye [7] Agbetoye *et al.* [8] Orhorhoro *et al.* [9]) for melon; Kamboj *et al.* [10] for pea; Also, the performance evaluation and optimization of post-harvest process such as dehulling and shelling of *moringa oleifera* seeds and some agricultural products using Response Surface Methodology (RSM), a statistical analysis tool in the modelling and optimization of more than two variables, to investigate the interactions between variables on selected responses have also been reported (Fadele and Aremu [2], Fadele and Aremu [3], Fakayode *et al.* [11]; Fakayode *et al.* [12], Fakayode and Ajav [13]) for moringa; Fakayode and Abobi [14], Sobowale *et al.* [15], Olayanju *et al.* [16] for orange peels, cocoyam noodles and paddy respectively.

However, the performance evaluation and optimization of depodding process of moringa to the best knowledge of the authors have not been reported. It was on this basis, Ikubanni *et al.* [17] designed and fabricated a moringa depodding/dehulling machine in other to mitigate the problems of the traditional manual depodding. However, the literature is sparse on the effects of process factors on the performance of the moringa depodding machine, interaction effects of the process factors and determination of their optimal settings. This study, therefore, focuses on the performance evaluation and optimization of a *Moringa Oleifera* Depodding Machine using a Response Surface Approach.

## 2 Materials and methods

### 2.1 Sample collection and preparation

The moringa pods used for this experiment was harvested from the moringa tree domiciled in the Teaching and Research farm of Landmark University (latitude 8° 9°0″ N, longitude 5° 61° 0″ E), Omu-Aran, Kwara state in June 2019. It was sorted out from already split pods as these will affect the performance of the machine. The initial moisture content of the moringa pods was determined using the AOAC [18] method to be 10.10 ± 0.3 % (wet basis). The materials were divided into 3 and further sub-divided into 3 replicates, two parts were conditioned into 8.20 ± 0.05 % and 9.09 ± 0.2 % wet basis respectively.

### 2.2 Experimental design

A 3^2^ factorial i-optimal randomized design was used for the experiment conducted. A total of 27 experiments were conducted with 3 replications (Table 1). The moisture content levels of 8.20, 9.09, and 10.10% wet basis were chosen based on the moisture content at harvest. The depodding drum rotational speeds used were 365, 487, and 564 rpm as reported by Ikubanni *et al.* [17].

**Table 1:** Moringa depodding output at various processing conditions.

Run	Moisture content (% db)	Speed of rotation (rpm)	TC (kg/hr)	ETC (kg/hr)	LR (man hour/kg)	DC (%)	CW	DE (%)	DK	UK	SBK (%)	BBK (%)
1	8.20	365.00	66.50	59.91	1.50	97.74	0.986	96.63	0.774	0.226	2.764	0.875
2	8.20	365.00	66.55	58.89	1.50	97.7	0.988	96.53	0.777	0.223	2.766	0.874
3	8.20	365.00	66.45	59.86	1.51	97.77	0.976	95.65	0.775	0.225	2.765	0.873
4	8.20	487.00	81.10	73.48	1.23	99.25	0.969	95.93	0.992	0.008	7.374	1.494
5	8.20	487.00	81.05	73.56	1.234	99.29	0.963	95.63	0.993	0.007	7.371	1.492
6	8.20	487.00	81.15	73.42	1.232	99.16	0.965	95.73	0.992	0.0084	7.374	1.49
7	8.20	584.00	133.00	120.91	0.75	100.00	0.953	95.30	1.00	0.00	14.20	1.546
8	8.20	584.00	133.50	120.78	0.75	99.9	0.949	94.81	1.00	0.00	14.23	1.548
9	8.20	584.00	133.23	120.97	0.75	99.8	0.954	95.21	1.00	0.00	14.30	1.56
10	9.09	365.00	61.41	55.48	1.628	94.52	0.991	94.10	0.69	0.31	2.01	0.655
11	9.09	365.00	61.45	55.36	1.627	94.24	0.993	93.54	0.68	0.32	2.11	0.657
12	9.09	365.00	61.32	55.53	1.631	94.58	0.992	93.84	0.688	0.312	2.14	0.652
13	9.09	487.00	77.31	71.31	1.29	97.21	0.973	94.4	0.98	0.02	6.48	1.38
14	9.09	487.00	77.24	71.23	1.295	97.18	0.974	94.67	0.977	0.023	6.47	1.384
15	9.09	487.00	77.37	71.45	1.292	97.25	0.975	94.87	0.978	0.022	6.5	1.381
16	9.09	584.00	122.34	113.24	0.82	98.61	0.964	95.4	0.99	0.01	12.56	1.471
17	9.09	584.00	122.19	113.12	0.818	98.64	0.966	95.25	0.988	0.012	12.59	1.47
18	9.09	584.00	122.30	113.35	0.818	98.65	0.965	95.25	0.987	0.013	12.54	1.45
19	10.10	365.00	52.34	52.34	1.91	90.11	0.998	89.8	0.57	0.43	1.52	0.46
20	10.10	365.00	52.31	52.32	1.912	90.15	0.996	89.84	0.568	0.432	1.5	0.456
21	10.10	365.00	52.45	52.43	1.907	90.19	0.997	89.93	0.567	0.433	1.54	0.462
22	10.10	487.00	73.52	67.42	1.36	94.68	0.984	93.5	0.95	0.05	5.26	1.12
23	10.10	487.00	73.45	67.48	1.361	94.73	0.985	93.28	0.948	0.052	5.28	1.125
24	10.10	487.00	73.67	67.36	1.357	94.69	0.986	93.37	0.952	0.048	5.267	1.14
25	10.10	584.00	112.45	109.35	0.89	95.86	0.975	93.6	0.97	0.03	10.56	1.21
26	10.10	584.00	112.23	109.30	0.891	95.88	0.974	93.41	0.969	0.031	10.568	1.24
27	10.10	584.00	112.56	109.43	0.816	95.87	0.976	93.6	0.97	0.03	10.55	1.23

TP, throughput capacity; ETP, effective throughput capacity; LR, labour requirement; DC, percentage depodded; CW, percentage wholeness; DE, depodding efficiency; DK, depodded kernel; UK, undepodded kernel; SBK, small broken kernel; BBK, big broken kernel.

### 2.3 Experimental procedures

**Figure 2:**
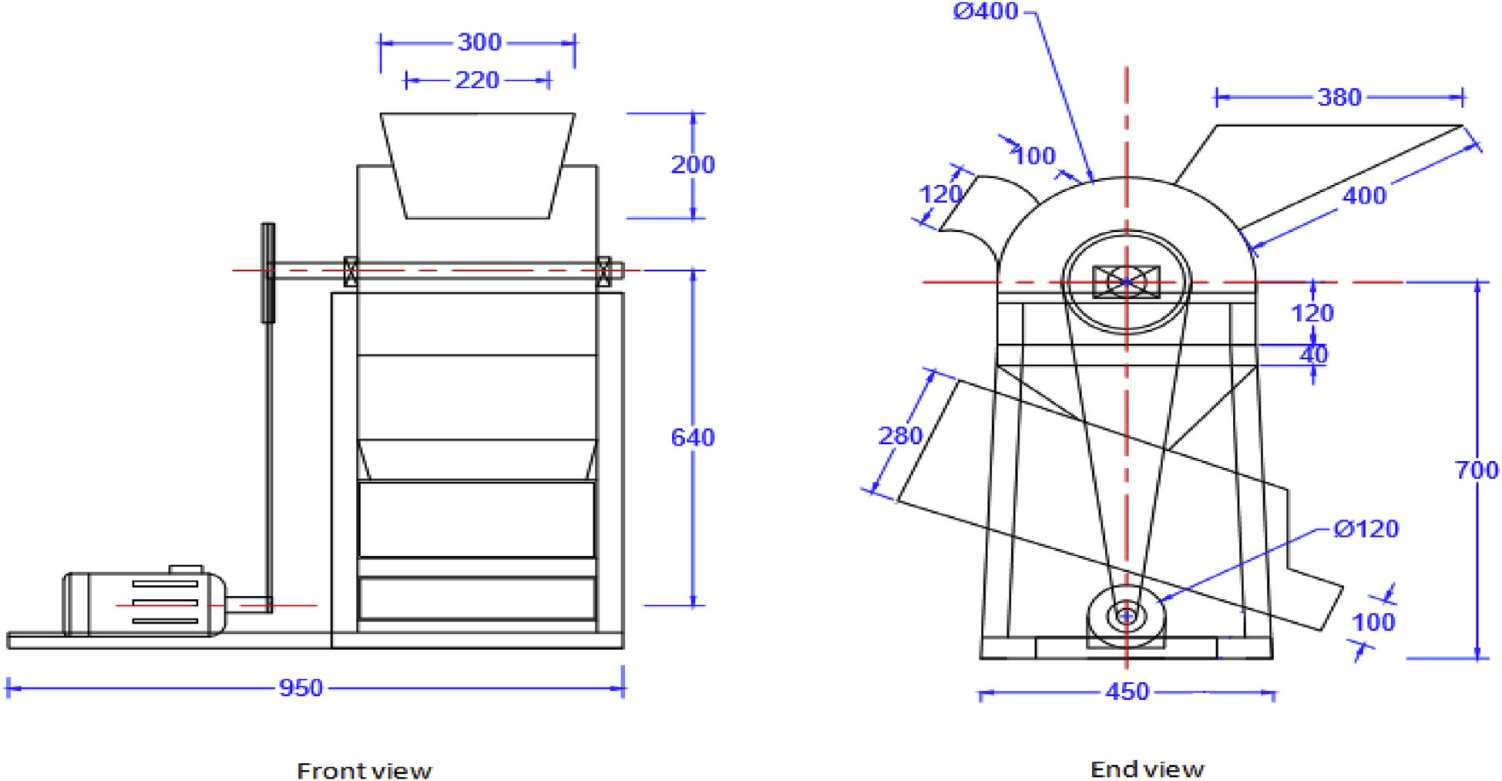
Front and end view of the developed moringa depodding machine.

**Figure 3:**
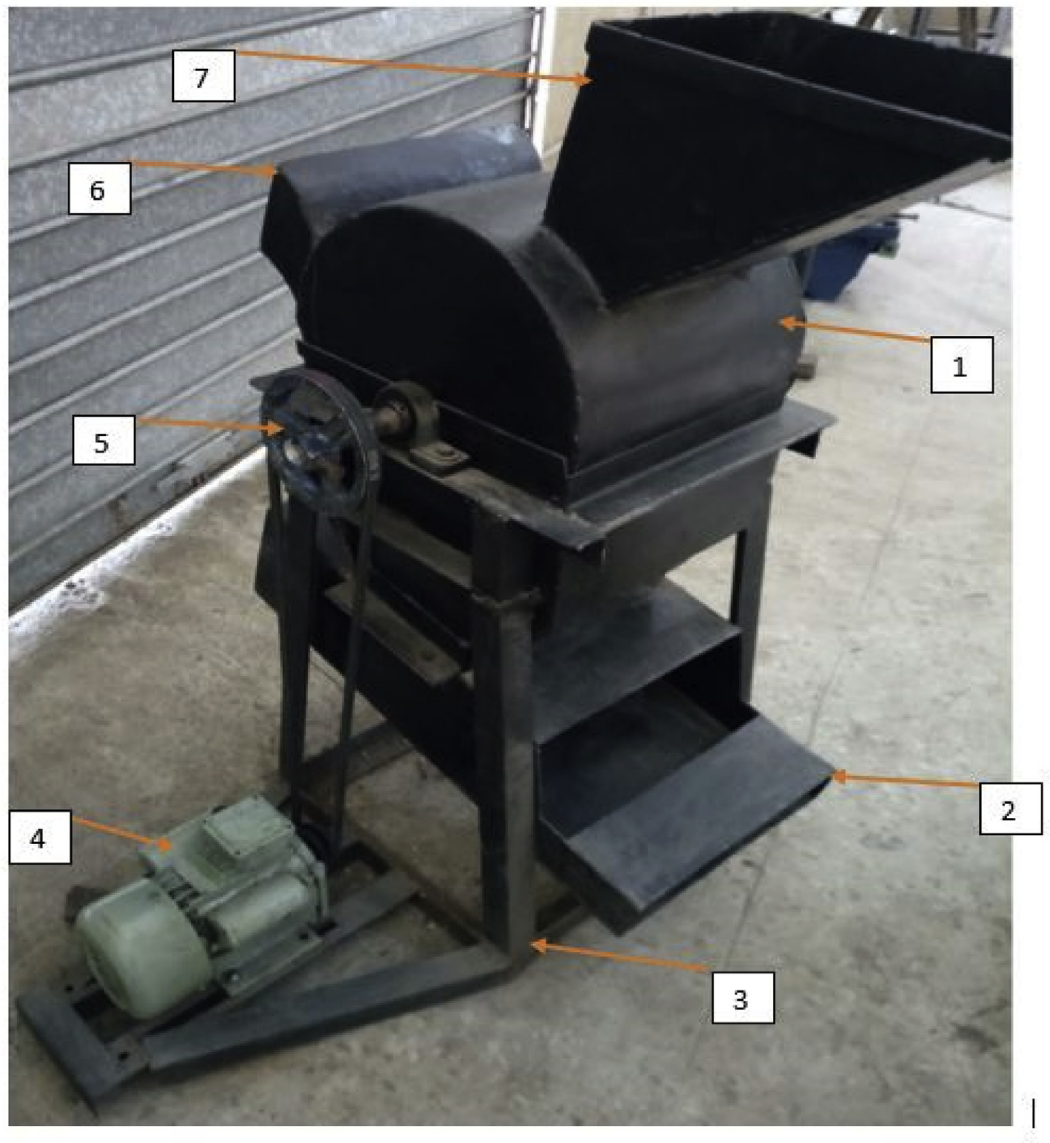
Pictorial view of the developed moringa depodding/dehulling machine. 1. Depoding drum cover 2. Seeds outlet 3. Supporting frame 4. Electric motor 5. Depoding drum pulley 6. Chaff oulet 7. Hopper.

The sorted moringa pods were fed into the moringa depodding machine at different moisture content. A constant feed rate of 5 pods per throw was used during the evaluation. The speed of rotation of the depodding drum was varied with the use of different pulley ratio. The machine used consists of a hopper, deppodding drum, concave, depodding unit casing, chaff and good product outlet, frame as shown in Figures 2 and 3. It was powered by a 1 hp electric motor Ikubanni *et al.* [17] Detailed specifications of the depodding machine are presented in Table 2.

**Table 2:** Machine specifications of the developed moringa deppoding machine.

S/N	Design element	Value	Unit
1.	Electric motor speed	1400	rpm
2.	Depodding shaft speed	487	rpm
3.	Weight of depodding drum	12.75	N
4.	Depodding shaftdiameter	30	Mm
5.	Depodding shaft length	500	Mm
6.	Angle of pulley groove	45	O
7.	Center to center distance of the pulley	640	mm
8.	Coefficient of friction	0.11	
9.	Plate thickness	2.5	Mm
10	Number of pulley groove required	1	
11	Bending moment of depodding shaft	1250	Nm
12	Torque transmitted to the depodding shaft	5.2	Nm

Source: Ikubanni et al. [17].

### 2.4 Performance evaluation

The performance evaluation of the developed moringa depodding machine was carried out using the equations suggested by Hussain *et al.* [19] and Okonkwo *et al.* [20].

(1) $$\text{Throughput}\;\text{capacity}\left(\frac{\text{Kg}}{\text{hr}}\right)=\frac{\text{Total}\;\text{weight}\;\text{of}\;\text{moringa}\;\text{subjected}\;\text{to}\;\text{depodding}}{\text{Time}\;\text{of}\;\text{operation}}$$

(2) $$\text{Effective}\;\text{throughput}\;\text{capacity}\left(\frac{\text{Kg}}{\text{hr}}\right)=\frac{\text{Actual}\;\text{weight}\;\text{of }\;\text{depodded}\;\text{moringa}}{\text{Effective}\;\text{operating}\;\text{time}}$$

(3) $$\text{Labour}\;\text{requirement}\left(\text{man}-\text{hour}\;\text{required}\;\text{per}\;\text{Kg}\right)=\frac{1}{\text{Throughput}\;\text{capacity}}$$

(4) $$\text{Percentage}\;\text{depodded}=\frac{{\text{M}}_{\text{dm}}}{{\text{M}}_{\text{tm}}}\times 100$$

(5) $$\text{Percentage}\;\text{wholeness}=1-\frac{{\text{M}}_{\text{bm}}}{{\text{M}}_{\text{tm}}}$$

(6) $$\text{Depodding}\;\text{efficiency}=\left(1-\frac{{\text{M}}_{\text{um}}}{{\text{M}}_{\text{tm}}}\right)\left(1-\frac{{\text{M}}_{\text{bm}}}{{\text{M}}_{\text{tm}}}\right)\times 100$$

(7) $$\text{Percentage}\;\text{Undepodded }=\frac{\text{Weight}\;\text{of}\;\text{undepodded}\;\text{moringa}}{\text{Total}\;\text{weight}\;\text{of}\;\text{moringa}\;\text{pods}\;\text{subjected}\;\text{to}\;\text{depodding}}$$

(8) $$\text{Small}\;\text{broken}\;\text{kernel}=\frac{\text{weight}\;\text{of}\;\text{small}\;\text{broken}\;\text{kernels }\left(\frac{1}{4}\text{th}\;\text{to}\;\frac{1}{8}\text{th}\;\text{of }\;\text{ball}\;\text{size}\right)}{\text{weight}\;\text{of}\;\text{kernels}}$$

(9) $$\text{Big}\;\text{broken}\;\text{kernel}=\frac{\text{weight}\;\text{of}\;\text{broken}\;\text{kernel}\geq \frac{1}{4}\text{th}\;\text{of}\;\text{ball}\;\text{size}}{\text{weight}\;\text{of}\;\text{kernels}}$$

where $M_{cm}$ = mass of depodded moringa (kg); $M_{tm}$ = total mass of moringa pods fed into the machine per time (kg); $M_{bm}$ = mass of broken undehulled moringa seed (kg); $M_{um}$ = mass of undehulled moringa seed (kg); ${\text{M}}_{\text{dm}}$ = mass of depodded moringa pods (kg).

### 2.5 Optimization of the machine performance

The RSM tool (Design-Expert version 12.0.1.0) was utilized for the experimental design, analyses, and generation of model equations that depicts the various performance of the developed moringa depodding machine. The predicted results were compared with the experimental results obtained as suggested by Fakayode *et al.* [11]. The efficiencies of the moringa depodding machine with the variables were evaluated using linear, two-factor interaction (2FI), quadratic, and cubic models to see which model performed best as suggested by Fakayode *et al.* [11] and Falade and Aremu [2]. Analysis of variance was conducted using for the various performance to determine the adequacy of the developed models, significance, fitness as well as their interactions with the performance responses as pointed out by Falade and Aremu [2]. The p-value was also analyzed. Optimization of the variables used was further analyzed, maximizing the desired responses (Throughput capacity, effective throughput capacity, depoddding efficiency, and depodded kernel) and minimizing the undesired responses (Labour requirement, undepodded moringa, small broken kernel, and big broken kernel) [21]. SPSS window 22 software was used to analyze the tests between-subjects effects of the processing variables on the performance of the developed machine.

## 3 Results and discussions

### 3.1 Effects of the moisture content and speed of rotation on throughput capacity (TP), effective throughput capacity (ETP), and labour requirement (LR)

#### 3.1.1 Effect of moisture content on TP, ETP, and LR

**Figure 4:**
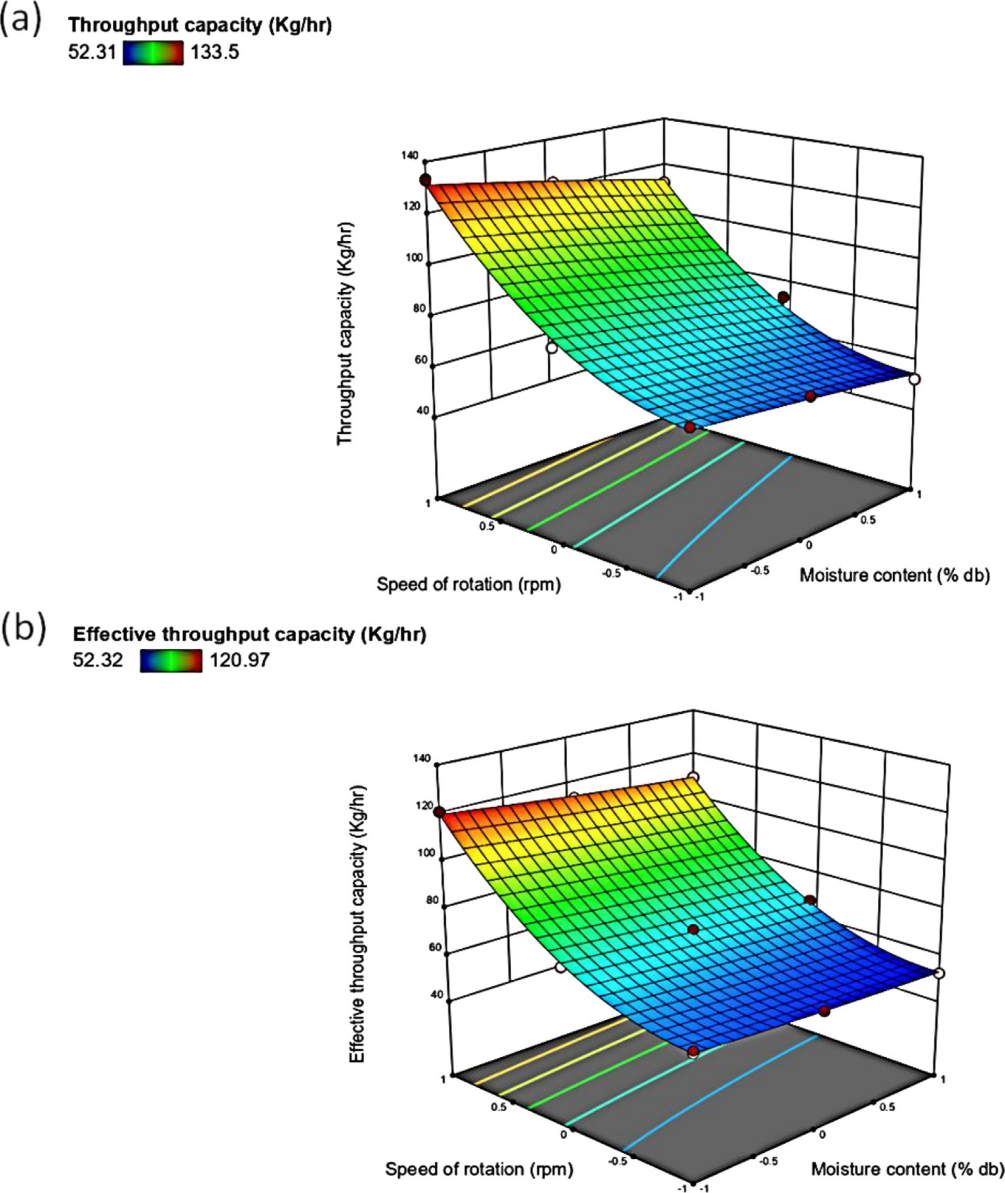
Response surface plot of moisture content cum speed of rotation on the (a) throughput capacity; (b) effective throughput capacity.

**Figure 5:**
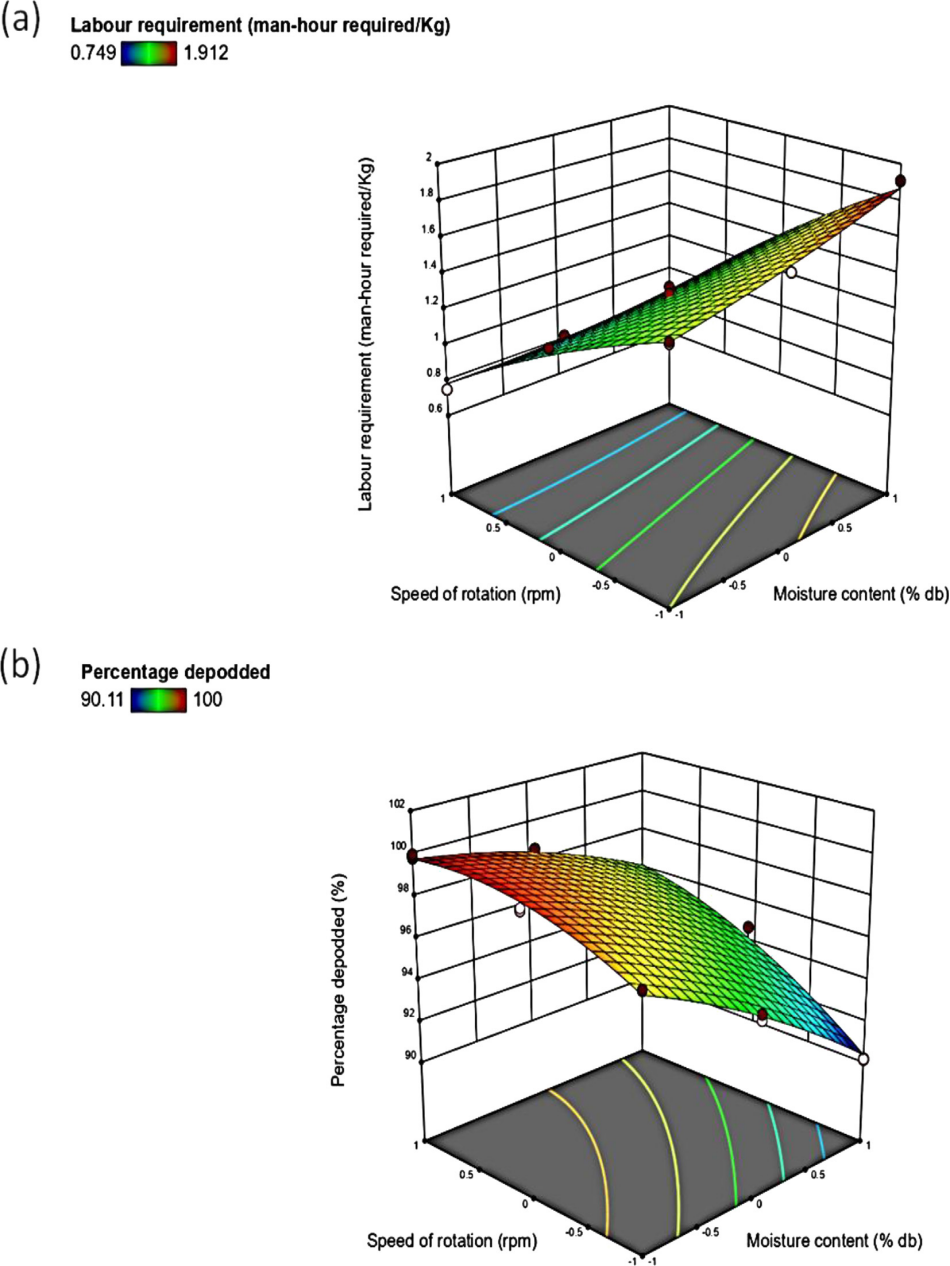
Response surface plot of moisture content cum speed of rotation on the (a) labour requirement; (b) depodding coefficient.

The increase in the moisture content slightly decreased the TP from133 to 40 kg/hr and ETP from 120 to 40 kg/hr of the depodding machine (Figure 4a and b). This might be because at lower moisture content, moringa pod easily split. An increase in moisture content caused the LR to increase from 0.8 to1.8 man-hours required per Kg (Figure 5a). Increased moisture content reduces the TP, thereby increasing the LR. These observations are in agreement with Falade and Aremu [2], a decreased TP with increased moisture content at 90^o^ bar inclination but fluctuate using other bar inclination for an impact type moringa shelling device.

#### 3.1.2 Effect of speed of rotation on TP, ETP, and LR

It was observed that the increase in the speed of rotation increased the TP and ETP of the depodding machine to 133 kg/h and 120 kg/h respectively (Figure 4a and b). This is might be due to at high speed of rotation, the spikes on the drum have a higher number of impacts with the pods therein. Iyanda *et al.* [5] reported an increased TP for a cocoa depodding machine with an increase in the speed of the depodding mechanism. The increase in the speed of rotation led to decrease in LR of the machine from 1.4 to 0.8 (Figure 5a). The LR is an inverse function of the TP, at a higher speed of rotation less time is required for completion of the depodding operation, and it leads to a higher TP and a lower LR. This observation is in concomitance with that reported by Okonkwo *et al.* [20], in which it was also reported that an increase in speed decreased the LR for a locust bean dehuller. Hussain *et al.* [19] also reported that using power-operated walnut crackers required the least LR as compared to the manual and hand-operated crackers.

#### 3.1.3 Interactive effect of moisture content and speed of rotation on TP, ETP, and LR

The interactive effect of the moisture content and speed of rotation increased the TP from 40 to 118 kg/hr and ETP from 40 to 115 kg/hr of the moringa depodding machine since the speed of rotation had a higher significant effect on the TP than the moisture content (Figure 4a, b). It is also expected since the depodding operation is achieved by the impact. The interactive effect of moisture content and speed of rotation showed that increased speed of rotation and moisture content decreased the LR from 1.2 to 0.8 man-hour required/Kg (Figure 5a). This finding is in support of Okonkwo *et al.* [20] who reported a similar increase in the TP of a locust beans dehuller with the interactive effect of increased speed of dehulling and decreased moisture content.

### 3.2 Effects of the moisture content and speed of rotation on percentage depodded (DC), coefficient of wholeness (CW), and the depodding efficiency (DE)

#### 3.2.1 Effect of moisture content on DC, CW, and DE

**Figure 6:**
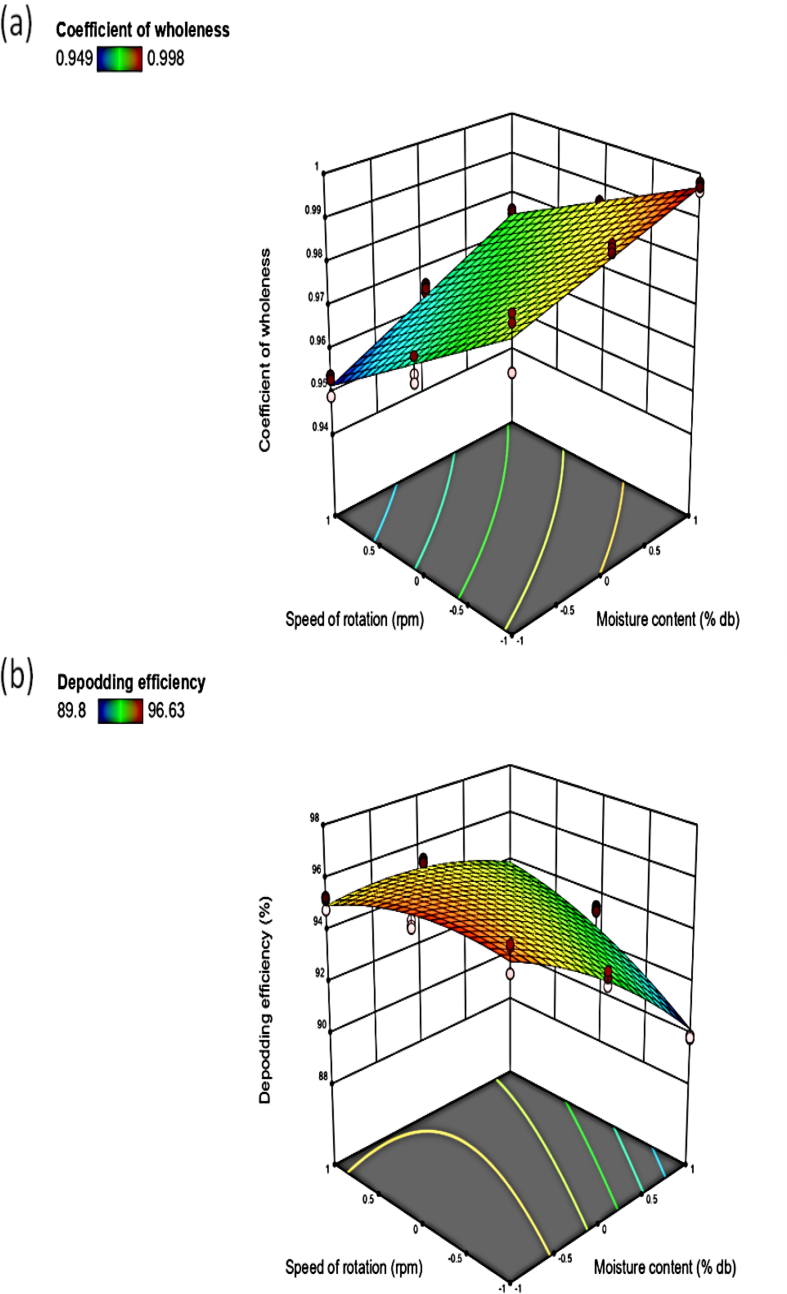
Response surface plot of moisture content cum speed of rotation on the (a) coefficient of wholeness; (b) depodding efficiency.

As indicated in Figure 5b, increased moisture content decreased the DC to 90%. At higher moisture content, the splitting of the moringa pod becomes more difficult due to the tough outer casing. This does not agree with Figueiredo *et al.* [22], it was revealed increased dehulling ability with increased moisture content for safflower seeds. But it agrees with Figueiredo *et al.* [23], it was reported decreased in the dehulling ability for confectionary sunflower seeds in a dehulling system with an increase in the moisture content. It can be seen from Figure 6a that increasing the moisture content increased the CW to 1. At higher moisture content less mechanical damage is encountered by the un-dehulled seeds due to toughness of the pod. A similar phenomenon was reported by Figueiredo *et al.* [22] for the percentage of the whole kernel as a function of moisture content for confectionary sunflower seeds in a dehulling system. Falade and Aremu [24] reported that the percentage whole kernel increased with an increase in the moisture content of un-dehulled moringa seeds during shelling operation for moringa. Increased moisture content decreased the DE to 90 % (Figure 6b). High moisture content makes splitting of the moringa pods difficult, thereby reducing the efficiency of the machine during the operation. Falade and Aremu [24] observed that the shelling efficiency of a moringa shelling device decreased with an increase in moisture content to 25 % wet basis, but increased afterward.

#### 3.2.2 Effect of speed of rotation on DC, CW, and DE

It was observed that the increase in the rotational speed, increased DC of the un-dehulled moringa seeds to 100% (Figure 5b). Increased speed of rotation increases the revolution and number of the impact of the depodding drum on the moringa pods in the depodding unit. These findings are not in agreement with the observation made by Okonkwo *et al.* [20], in which it was reported that increase in speed resulted in decreased quantitative dehulling efficiency; Hussain *et al.* [19] described that using a power-operated cracker had the least cracking coefficient as compared to the hand and manually operated cracker; Figueiredo *et al.* [22] revealed increased dehulling ability with increased peripheral speed for safflower seeds. An increase in the speed of rotation decreased the CW of the un-dehulled moringa seeds to 0.95 (Figure 6a). The higher speed of rotation increases the impact made on the pod during operation thereby increasing the mechanical damage on the un-dehulled seeds. Similar phenomenon was reported by Figueiredo *et al.* [22] for the percentage of the whole kernel as a function of peripheral speed for confectionary sunflower seeds in a dehulling system; Okonkwo *et al.* [20] revealed that increased speed of rotation decreased the coefficient of the wholeness of locust bean in a dehuller; Sharma *et al.* [25] also reported that the increased speed of a centrifugal impact-type decorticator increased the percentage of the whole kernel of Tung fruits from 1600 to 1800 rpm but decreased from 1800 to 2000 rpm. Increase speed of rotation increased the DE of the machine to 95 %, but a slight decrease was noticed for the DE from the speed of 500–584 rpm (Figure 6b). At the high-speed rate, the number of the impact of the depodding drum on the pods therein increases, in which the DE increases. The observed result was in tandem with Oloko and Agbetoye [7] for the depodding efficiency of melon seeds which increased with an increase in the speed of the machine; Iyanda *et al.* [5] reported a decrease in DE for the cocoa depodding machine with an increase in speed; Falade and Aremu [2] delineated that the shelling efficiency of a moringa shelling device increased with an increase in speed; Okonkwo *et al.* [20] reported a decreased qualitative dehulling efficiency with an increase in speed for a locust bean dehulling machine.

#### 3.2.3 Interactive effect of moisture content and speed of rotation on DC, CW, and DE

The interactive effect of the moisture content and the speed of rotation revealed that increased moisture content with speed of rotation increased the depodding coefficient from 95 to 96 % (Figure 5b). A similar result was reported by Figueiredo *et al.* [23] for the dehulling ability for confectionary sunflower seeds in a dehulling system. The interactive effect of factors showed that increased moisture content and speed of rotation resulted in a decreased CW from 0.965 to 0.975for the undehulled moringa seeds during its operation (Figure 6a). This observation was also delineated by Figueiredo *et al.* [22] for the percentage of the whole kernel as a function of moisture content with peripheral speed for confectionary sunflower seeds in a dehulling system. The interactive effect of moisture content and speed of rotation showed that increased speed and decreased moisture content increased the DE from 93 to 94 %of the developed machine (Figure 6b). A similar result was reported by Fakayode *et al.* [11] for the dehulling efficiency of moringa pods to moisture content and speed.

### 3.3 Effects of the moisture content and speed of rotation on percentage undepodded (UK)

#### 3.3.1 Effect of moisture content on UK

**Figure 7:**
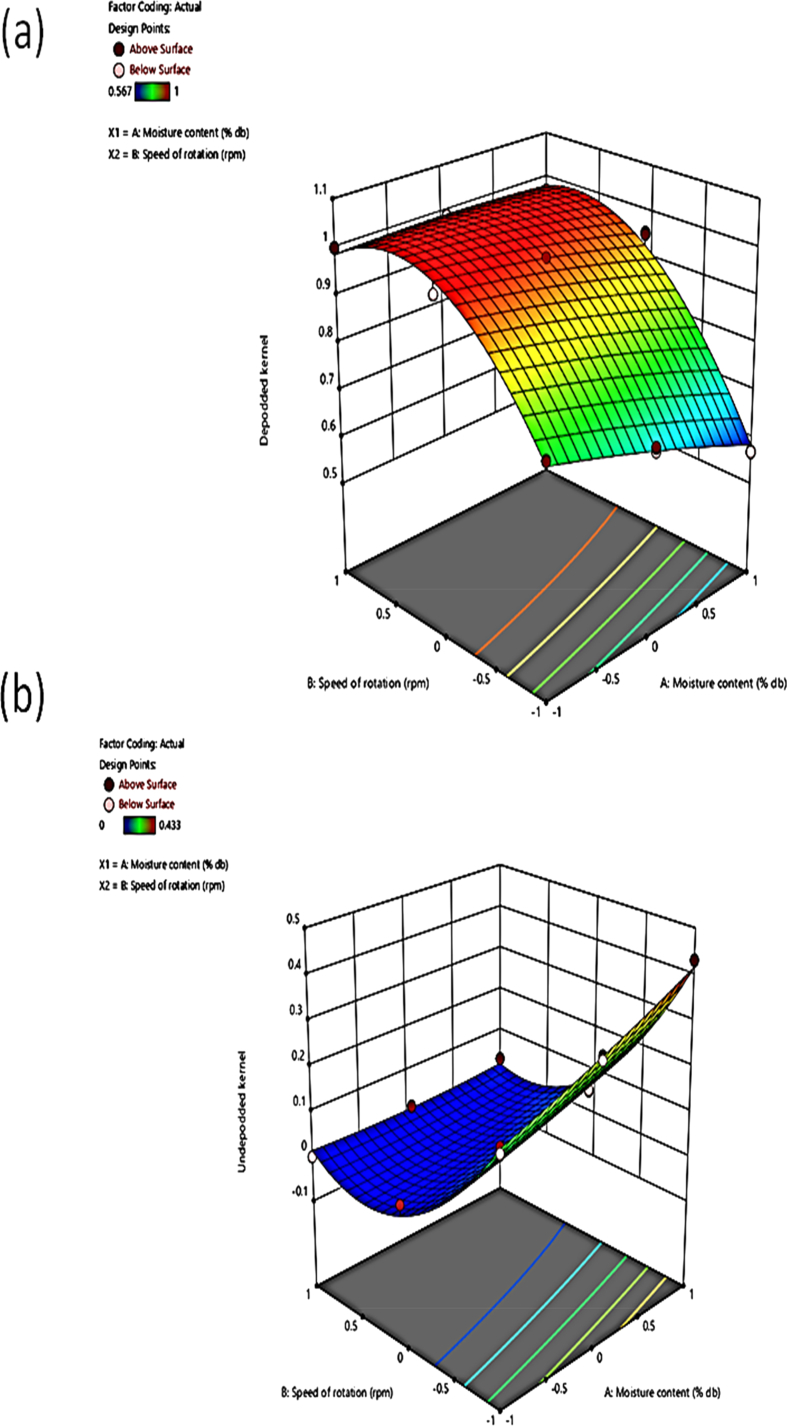
Response surface plot of moisture content cum speed of rotation on the (a) depodded kernel (b) undepodded kernel.

Increased moisture content increased the UK to 0.4 (Figure 7b). This might be due to the tough outer coat of the moringa pod at high moisture content. Similar results were reported by Sharma *et al.* [25] increased moisture content increased the percentage of unshelled Tung fruits. Aremu *et al.* [26] reported that increased moisture content of jatropha seeds in a jatropha shelling device decreased the percentage unshelled kernel but from 11% moisture content wet basis afterward the percentage unshelled kernel increased.

#### 3.3.2 Effect of speed of rotation on UK

Increased speed of rotation decreased the UK to 0 (Figure 7b). This might be because, at higher speed, more pods split due to the high frequency of impact. Similar results were reported by Sharma *et al.* [25] increased speed reduced the percentage of unshelled Tung fruits during the shelling.

#### 3.3.3 Interactive effect of moisture content and speed of rotation on UK

The interactive effect of moisture content and speed of rotation on the UK showed that a simultaneous increase in speed and moisture content decreased the UK from 0.1 to 0.01 (Figure 7b).

### 3.4 Effects of the moisture content and speed of rotation on small broken kernel (SBK) and big broken kernel (BBK)

#### 3.4.1 Effect of moisture content on SBK and BBK

**Figure 8:**
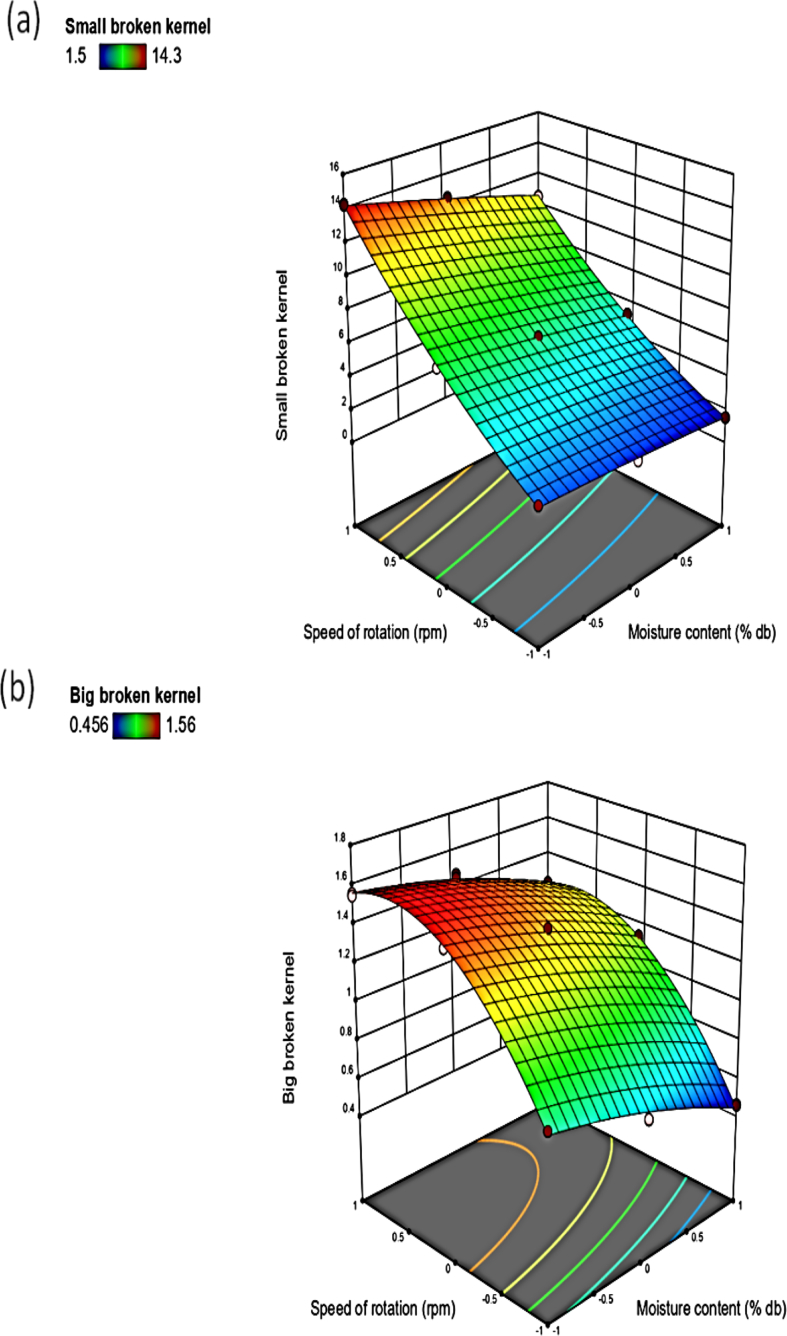
Response surface plot of moisture content cum speed of rotation on the (a) small broken kernel; (b) big broken kernel.

From Figure 8a, b, increased moisture content decreased the BBK to 0.4%, but a stable trend of 2% was observed for SBK. At higher moisture content the undehulled moringa seeds were shielded by the pod and outer coat, so there was less mechanical damage. Falade and Aremu [27] reported that percentage broke at 90^o^ cylinder bar inclination reduced with increased moisture content to18% but increased afterward for moringa in a shelling device. Falade and Aremu [24] revealed that the broken kernel increased from 8 to 11.3% moisture content but decreased afterward for moringa in a shelling machine.

#### 3.4.2 Effect of speed of rotation on SBK and BBK

Increased speed of rotation increased the SBK to 12% and the BBK to 1.6% (Figure 8a, b). At a higher speed, there was more mechanical damage caused to the product due to the increased impact of the rotating drum. These results are in agreement with the result reported by Iyanda *et al.* [5], in which it was revealed that mechanical damage caused by a cocoa depodding machine increased with increased speed.

#### 3.4.3 Interactive effect of moisture content and speed of rotation on SBK and BBK

The interactive effects of the moisture content and the speed of rotation showed a simultaneous increase in moisture content with speed increased the BBK and the SBK. Similar results were also reported by Figueiredo *et al.* [22], in which it was revealed that increased moisture content with peripheral speed for safflower seeds increased the percentage fines.

### 3.5 Modelling of the performance for the developed depodding machine

Quadratic and 2FI models were individually adapted for the prediction of the performance of the machine. The following response equations were generated:

(11) $$\text{TP}=77.63-7.09\text{MC}+31.28\text{SR}-1.67\text{MC}\times \text{SR}-0.46{\text{MC}}^{2}+14.05{\text{SR}}^{2}$$

(12) $$\text{ETP}=70.41-4.13\text{MC}+29.35\text{SR}-1.08\text{MC}\times \text{SR}+0.50{\text{MC}}^{2}+14.40{\text{SR}}^{2}$$

(13) $$\text{LR}=1.28+0.11\text{MC}-0.43\text{SR}-0.07\text{MC}\times \text{SR}+0.02{\text{MC}}^{2}-0.05{\text{SR}}^{2}$$

(14) $$\text{DC}=97.38-2.69\text{MC}+2.01\text{SR}+0.89\text{MC}\times \text{SR}-0.50{\text{MC}}^{2}-0.93{\text{SR}}^{2}$$

(15) CW=0.98+0.01MC−0.01SR+0.002MC×SR

(16) $$\text{DE}=95.00-1.73\text{MC}+0.67\text{SR}+1.21\text{MC}\times \text{SR}-0.61{\text{MC}}^{2}-0.61{\text{SR}}^{2}$$

(17) $$\text{UK}=0.02+0.05\text{MC}-0.15\text{SR}-0.04\text{MC}\times \text{SR}+0.01{\text{MC}}^{2}+0.14{\text{SR}}^{2}$$

(18) $$\text{SBK}=6.43-1.17\text{MC}+5.17\text{SR}-0.61\text{MC}\times \text{SR}-0.09{\text{MC}}^{2}+0.91{\text{SR}}^{2}$$

(19) $$\text{BBK}=1.36-0.18\text{MC}+0.38\text{SR}+0.02\text{MC}\times \text{SR}-0.05{\text{MC}}^{2}0.30{\text{SR}}^{2}\;$$

where MC = moisture content % wet basis; SR = speed of rotation; TP = throughput capacity (Kg/hr); ETP = effective throughput capacity (kg/hr); LR = labour requirement (man-hour required per kg); DC = percentage depodded; CW = coefficient of wholeness; DE = depodding efficiency; UK = percentage undepodded kernel; SBK = small broken kernel; BBK = big broken kernel.Model F-values for the TP, ETP, LR, DC, CW, DE, UK, SBK, and BBK were 783.14, 3725.13, 503.86, 464.73, 206.30, 76.49, 427.15, 10254.12, and 1431.66 respectively (Table 3), indicating significant models with only a 0.01% chance that an F-value this large could occur due to noise. Significant model terms are indicated by p > F less than 0.05. For TP, ETP, LR, and UK (MC, SR, MC x SR, and SR^2^) are the significant model terms, for DC, DE, SBK, and BBK (MC, SR, MC x SR, MC^2^, and SR^2^) are the significant terms and for CW (MC, SR, and MC x SR) are the significant terms (Table 3). It was noticed that the moisture content and speed have a significant effect on the performance. The “Lack of Fit F-values” for the TP, ETP, LR, DC, CW, DE, UK, SBK, and BBK were 2462.19, 142.23, 42.29, 106.94, 1.51, 18.96, 424.14, 66.50, and 51.56 respectively (Table 3). The “Lack of Fit F-values” was only a 23.71 % chance for CW and a 0.01 % chance for other performance efficiencies. Lack of Fit F-values was not significant for CW but others were significant. Adeq precision determines the ratio of signal to noise, and a value greater than 4 is desirable. The Adeq precision ratios for TP, ETP, LR, DC, CW, DE, UK, SBK, and BBK were 72.51, 148.37, 60.16, 64.94, 42.13, 26.96, 54.28, 268.40, and 104.53 respectively (Table 4) showing an adequate signal required for navigating within the design space. The R^2^ values for the TP, ETP, LR, DC, UK, SBK, BBK was 0.99, while for CW and DE it was 0.96 and 0.95 (Table 4), indicating high correlation value. The summary of the ANOVA indicates that the speed of rotation had the highest effect on the performance efficiency of the depodding machine developed as compared to the moisture content. The linear terms showed the highest significance. In predicting TP, ETP, LR, DC, UK, SBK, BBK, and DE, a quadratic model was selected while CW 2FI was selected based on the evaluation parameters (Table 4). Similar findings were reported by Fakayode *et al.* [11] and Shittu and Ndrika [28]. Table 5 presented the test between-subjects effects of the moisture content and speed of rotation on the performance efficiencies (TP, ETP, LR, DC, CW, DE, UK, SBK, and BBK). For the various performance efficiencies, the moisture content and speed of rotation are significant. The interaction between moisture content and the speed of rotation was also significant, except for CW. This signifies that the variables must be properly controlled as they affect the performance of the process.

**Table 3:** Model selection for the performance efficiency of the developed Moringa depodding machine.

	Throughput capacity				Effective throughput capacity			
	Linear	2FI	Quadratic	Cubic	Linear	2FI	Quadratic	Cubic
SD	7.43	7.49	2.24	0.22	7.30	7.41	0.96	0.58
R^2^	0.93	0.94	0.99	1.00	0.93	0.93	0.99	0.99
Mean	86.68	86.68	86.68	86.68	80.34	80.34	80.34	80.34
Adj. R^2^	0.93	0.93	0.99	0.99	0.92	0.92	0.99	0.99
C.V.	8.57	8.64	2.59	0.26	9.08	9.23	1.19	0.73
Pred. R^2^	0.92	0.92	0.99	0.99	0.91	0.90	0.99	0.99
PRESS	1623.15	1662.67	172.18	1.80	1575.76	1660.00	31.02	12.03
Adeq. Prec.	30.98	26.61	72.51	664.45	27.52	23.46	148.37	215.28
	Labour requirement				Percentage depodded			
	Linear	2FI	Quadratic	Cubic	Linear	2FI	Quadratic	Cubic

SD	0.07	0.05	0.04	0.02	0.87	0.61	0.31	0.13
R^2^	0.97	0.99	0.99	0.99	0.92	0.96	0.99	0.99
Mean	1.26	1.26	1.26	1.26	96.43	96.43	96.43	96.43
Adj. R^2^	0.97	0.99	0.99	0.99	0.91	0.96	0.99	0.99
C.V.	5.40	3.63	3.03	1.29	0.90	0.64	0.32	0.13
Pred. R^2^	0.96	0.98	0.99	0.99	0.89	0.95	0.99	0.99
PRESS	0.15	0.06	0.05	0.01	23.92	12.04	3.21	0.56
Adeq. Prec.	47.76	61.60	60.16	130.86	32.48	39.91	64.94	143.26
	Percentage wholeness				Depodding efficiency			
	Linear	2FI	Quadratic	Cubic	Linear	2FI	Quadratic	Cubic
SD	0.003	0.003	0.003	0.003	1.05	0.63	0.47	0.32
R^2^	0.95	0.96	0.97	0.97	0.70	0.90	0.95	0.98
Mean	0.98	0.98	0.98	0.98	94.19	94.19	94.19	94.19
Adj. R^2^	0.95	0.96	0.97	0.96	0.67	0.88	0.94	0.97
C.V.	0.33	0.29	0.26	0.28	1.12	0.67	0.50	0.34
Pred. R^2^	0.94	0.95	0.95	0.94	0.59	0.85	0.91	0.96
PRESS	0.0003	0.0003	0.0002	0.0003	35.90	13.09	7.55	3.84
Adeq. Prec.	42.59	42.13	37.28	30.53	13.62	24.32	26.96	36.75
	Percentage Undepodded							
	Linear	2FI	Quadratic	Cubic				
SD	0.08	0.07	0.02	0.002				
R^2^	0.76	0.79	0.99	0.99				
Mean	0.12	0.12	0.12	0.12				
Adj. R^2^	0.74	0.77	0.99	0.99				
C.V.	65.44	61.46	13.99	1.77				
Pred. R^2^	0.70	0.73	0.98	0.99				
PRESS	0.19	0.17	0.01	0.0002				
Adeq. Prec.	15.22	14.03	54.28	368.93				
	Small broken kernel				Big broken kernel			
	Linear	2FI	Quadratic	Cubic	Linear	2FI	Quadratic	Cubic
SD	0.64	0.48	0.10	0.04	0.15	0.15	0.02	0.01
R^2^	0.98	0.99	0.99	0.99	0.85	0.85	0.99	0.99
Mean	6.98	6.98	6.98	6.98	1.14	1.14	1.14	1.14
Adj. R^2^	0.98	0.99	0.99	0.99	0.83	0.83	0.99	0.99
C.V.	9.12	6.86	1.43	0.60	13.36	13.57	2.00	1.16
Pred. R^2^	0.98	0.99	0.99	0.99	0.82	0.81	0.99	0.99
PRESS	12.63	6.81	0.34	0.06	0.67	0.71	0.02	0.01
Adeq. Prec.	59.70	68.76	268.40	557.85	22.10	18.84	104.53	152.69

Abbreviations: SD, standard deviation; C.V., coefficient of variation; Adj. R^2^, adjusted R^2^; Pred. R^2^, predicted R^2^; PRESS, predicted residual sum of squares; Adeq. prec., adequate precision.

**Table 4:** ANOVA for response surface models for the performance efficiency of the developed moringa depodding machine.

Throughput capacity (Quadratic)					
Source	SS	Df	MS	F-value	p > F
Model	19733.44	5	3946.69	783.14	<0.0001
A	903.83	1	903.83	179.35	<0.0001
B	17610.64	1	17610.64	3494.49	<0.0001
AB	33.63	1	33.63	6.67	0.0173
A^2^	1.29	1	1.29	0.26	0.6180
B^2^	1184.04	1	1184.04	234.95	<0.0001
Residual	105.83	21	5.04	-	-
Lack of fit	105.57	3	35.19	2462.19	<0.0001
Pure Error	0.26	18	0.01	-	-
Cor Total	19839.27	26	-	-	-
Effective throughput capacity (Quadratic)					
Source	SS	df	MS	F-value	p > F
Model	17073.78	5	3414.76	3725.13	<0.0001
A	307.11	1	307.11	335.02	<0.0001
B	15507.37	1	15507.37	16916.87	<0.0001
AB	14.11	1	14.11	15.39	0.0008
A^2^	1.52	1	1.52	1.66	0.21
B^2^	1243.68	1	1243.68	1356.72	<0.0001
Residual	19.25	21	0.92	-	-
Lack of fit	18.47	3	6.16	142.23	<0.0001
Pure Error	0.78	18	0.04	-	-
Cor Total	17093.03	26	-	-	-
Labour requirement (Quadratic)					
Source	SS	df	MS	F-value	p > F
Model	3.69	5	0.74	503.86	<0.0001
A	0.21	1	0.21	143.90	<0.0001
B	3.40	1	3.40	2320.23	<0.0001
AB	0.06	1	0.06	43.25	<0.0001
A^2^	0.003	1	0.003	2.25	0.15
B^2^	0.014	1	0.014	9.66	0.01
Residual	0.03	21	0.002	-	-
Lack of fit	0.03	3	0.009	42.29	<0.0001
Pure Error	0.004	18	0.0002	-	-
Cor Total	3.76	26	-	-	-
Depodding coefficient (Quadratic)					
Source	SS	df	MS	F-value	p > F
Model	219.38	5	43.88	464.73	<0.0001
A	130.41	1	130.41	1381.28	<0.0001
B	72.84	1	72.84	771.53	<0.0001
AB	9.49	1	9.49	100.49	<0.0001
A^2^	1.50	1	1.50	15.85	<0.0007
B^2^	5.15	1	5.15	54.51	<0.0001
Residual	1.98	21	0.09	-	-
Lack of fit	1.88	3	0.63	106.94	<0.0001
Pure Error	0.11	18	0.01	-	-
Cor Total	221.37	26	-	-	-
Coefficient of wholeness (2FI)					
Source	SS	df	MS	F-value	p > F
Model	0.0049	3	0.0016	206.30	<0.0001
A	0.0016	1	0.0016	199.68	<0.0001
B	0.0032	1	0.0032	410.91	<0.0001
AB	0.0001	1	0.0001	8.32	0.0084
Residual	0.0002	23	7.85E-06	-	-
Lack of fit	0.0001	5	0	1.51	0.24
Pure Error	0.0001	18	7.07E-04	-	-
Cor Total	0.005	26	-	-	-
Depodding efficiency (Quadratic)					
Source	SS	df	MS	F-value	p > F
Model	83.72	5	16.74	76.49	<0.0001
A	53.70	1	53.70	245.32	<0.0001
B	7.96	1	7.96	36.36	<0.0001
AB	17.59	1	17.59	80.37	<0.0001
A^2^	2.20	1	2.20	10.03	0.005
B^2^	2.27	1	2.27	10.37	0.004
Residual	4.60	21	0.22	-	-
Lack of fit	3.49	3	1.16	18.96	<0.0001
Pure Error	1.11	18	0.06	-	-
Cor Total	88.32	26	-	-	-
Depodded kernel (Quadratic)					
Source	SS	df	MS	F-value	p > F
Model	0.62	5	0.12	428.68	<0.0001
A	0.04	1	0.04	136.23	<0.0001
B	0.43	1	0.43	1501.08	<0.0001
AB	0.02	1	0.02	81.55	<0.0001
A^2^	0.0004	1	0.0004	1.43	0.24
B^2^	0.1215	1	0.12	423.11	<0.0001
Residual	0.01	21	0.0003	-	-
Lack of fit	0.01	3	0.002	424.59	<0.0001
Pure Error	0.0001	18	4.67E-06	-	-
Cor Total	0.62	26	-	-	-
Undepodded kernel (Quadratic)					
Source	SS	df	MS	F-value	p > F
Model	0.62	5	0.12	427.15	<0.0001
A	0.04	1	0.04	135.64	<0.0001
B	0.43	1	0.43	1496.01	<0.0001
AB	0.02	1	0.02	81.27	<0.0001
A^2^	0.004	1	0.0004	1.44	0.24
B^2^	0.12	1	0.12	421.42	<0.0001
Residual	0.006	21	0.0003	-	-
Lack of fit	0.006	3	0.002	424.14	<0.0001
Pure Error	0.0001	18	4.69E-06	-	-
Cor Total	0.62	26	-	-	-
Small broken kernel (Quadratic)					
Source	SS	df	MS	F-value	p > F
Model	514.58	5	102.92	10254.12	<0.0001
A	24.73	1	24.73	2464.13	<0.0001
B	480.32	1	480.32	47857.18	<0.0001
AB	4.46	1	4.46	444.53	<0.0001
A^2^	0.05	1	0.05	4.79	0.0401
B^2^	5.02	1	5.02	499.96	<0.0001
Residual	0.21	21	0.01	-	-
Lack of fit	0.19	3	0.06	66.50	<0.0001
Pure Error	0.02	18	0.001	-	-
Cor Total	514.79	26	-	-	-
Big broken kernel (Quadratic)					
Source	SS	df	MS	F-value	p > F
Model	3.69	5	0.74	1431.66	<0.0001
A	0.61	1	0.61	1179.87	<0.0001
B	2.54	1	2.54	4925.65	<0.0001
AB	0.006	1	0.006	11.78	0.003
A^2^	0.01	1	0.01	23.28	<0.0001
B^2^	0.52	1	0.52	1017.73	<0.0001
Residual	0.01	21	0.001	-	-
Lack of fit	0.01	3	0.003	51.56	<0.0001
Pure Error	0.001	18	0.0001	-	-
Cor Total	3.70	26	-	-	-

p > F less than 0.05 indicates model terms are significant; SS, sum of squares; df, degree of freedom; MS, mean square.

**Table 5:** Test of between-subjects effects of moisture content and speed of rotation on the various performance efficiencies for the developed moringa depodding machine.

Sources	Performance efficiency	Sum of squares	df	Mean square	F-value	Sig.
Corrected model	TP	19839.012^a^	8	2479.877	173507.815	.000
	ETP	17092.252^b^	8	2136.532	49355.195	.000
	LR	3.718^c^	8	.465	2188.194	.000
	DC	221.261^d^	8	27.658	4726.307	.000
	CW	.005^f^	8	.001	86.819	.000
	DE	87.210^g^	8	10.901	177.566	.000
	DK	.621^h^	8	.078	16640.079	.000
	UK	.621^i^	8	.078	16564.028	.000
	SBK	514.777^j^	8	64.347	66400.585	.000
	BBK	3.700^k^	8	.463	7376.547	.000
Intercept	TP	202876.274	1	202876.274	14194504.777	.000
	ETP	174287.990	1	174287.990	4026159.923	.000
	LR	43.014	1	43.014	202542.421	.000
	DC	251073.827	1	251073.827	42905021.028	.000
	CW	25.749	1	25.749	3639888.424	.000
	DE	239526.112	1	239526.112	3901547.433	.000
	DK	20.847	1	20.847	4467266.865	.000
	UK	.397	1	.397	84768.056	.000
	SBK	1317.252	1	1317.252	1359289.544	.000
	BBK	34.896	1	34.896	556516.849	.000
MC	TP	905.125	2	452.562	31664.116	.000
	ETP	308.630	2	154.315	3564.774	.000
	LR	.214	2	.107	504.107	.000
	DC	131.908	2	65.954	11270.613	.000
	CW	.002	2	.001	111.016	.000
	DE	55.895	2	27.948	455.230	.000
	DK	.040	2	.020	4234.056	.000
	UK	.039	2	.020	4211.580	.000
	SBK	24.780	2	12.390	12785.197	.000
	BBK	.620	2	.310	4946.318	.000
SR	TP	18794.680	2	9397.340	657497.241	.000
	ETP	16751.046	2	8375.523	193479.742	.000
	LR	3.413	2	1.707	8036.054	.000
	DC	77.989	2	38.994	6663.578	.000
	CW	.003	2	.002	230.717	.000
	DE	10.229	2	5.115	83.311	.000
	DK	.552	2	.276	59181.341	.000
	UK	.552	2	.276	58911.384	.000
	SBK	485.342	2	242.671	250415.435	.000
	BBK	3.064	2	1.532	24434.095	.000
MC×SR	TP	139.207	4	34.802	2434.952	.000
	ETP	32.576	4	8.144	188.132	.000
	LR	.090	4	.023	106.309	.000
	DC	11.365	4	2.841	485.519	.000
	CW	7.844E-5	4	1.961E-5	2.772	.059
	DE	21.085	4	5.271	85.862	.000
	DK	.029	4	.007	1572.460	.000
	UK	.029	4	.007	1566.574	.000
	SBK	4.655	4	1.164	1200.854	.000
	BBK	.016	4	.004	62.888	.000
Error	TP	.257	18	.014	-	-
	ETP	.779	18	.043	-	-
	LR	.004	18	.000	-	-
	DC	.105	18	.006	-	-
	CW	.000	18	7.074E-6	-	-
	DE	1.105	18	.061	-	-
	DK	8.400E-5	18	4.667E-6	-	-
	UK	8.437E-5	18	4.687E-6	-	-
	SBK	.017	18	.001	-	-
	BBK	.001	18	6.270E-5	-	-
Total	TP	222715.543	27	-	-	-
	ETP	191381.021	27	-	-	-
	LR	46.735	27	-	-	-
	DC	251295.193	27	-	-	-
	CW	25.754	27	-	-	-
	DE	239614.427	27	-	-	-
	DK	21.469	27	-	-	-
	UK	1.019	27	-	-	-
	SBK	1832.046	27	-	-	-
	BBK	38.597	27	-	-	-
Corrected Total	TP	19839.269	26	-	-	-
	ETP	17093.032	26	-	-	-
	LR	3.721	26	-	-	-
	DC	221.367	26	-	-	-
	CW	.005	26	-	-	-
	DE	88.315	26	-	-	-
	DK	.621	26	-	-	-
	UK	.621	26	-	-	-
	SBK	514.794	26	-	-	-
	BBK	3.701	26	-	-	-

R^2^ ≥ .975 (Adjusted R^2^ ≥ .964); p < 0.05, Significant; TP, throughput capacity; ETP, effective throughput capacity; LR, labour requirement; DC, depodding coefficient; CW, coefficient of wholeness; DE, depodding efficiency; DK, depodded kernel; UK, undepodded kernel; SBK, small broken kernel; BBK, big broken kernel; MC, moisture content; SR, speed of rotation.

### 3.6 Optimization

**Figure 9:**
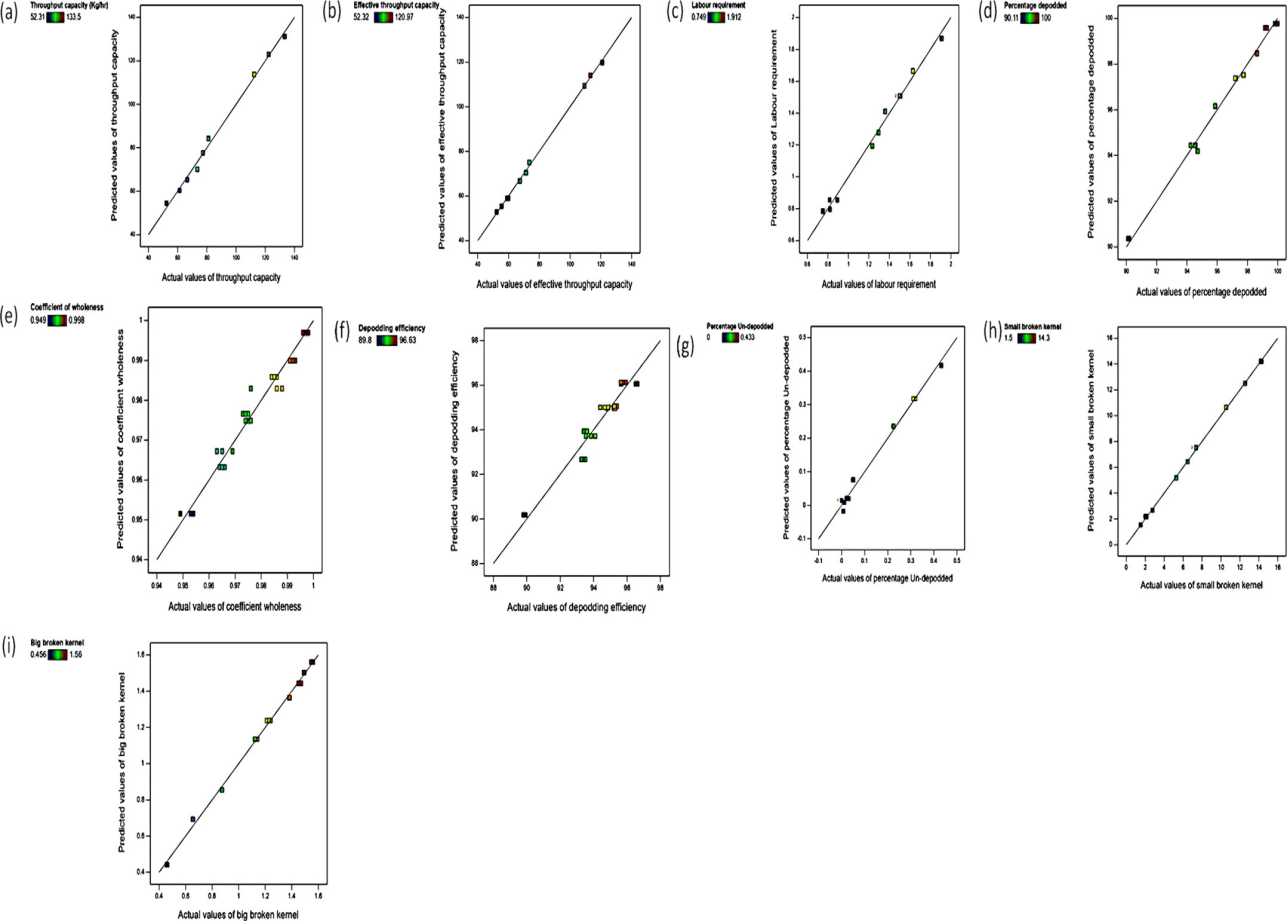
Comparison of the predicted and actual values of the performance efficiency of the developed depodding machine; (a) throughput capacity; (b) effective throughput capacity; (c) labour requirement; (d) depodding coefficient; (e) coefficient of wholeness; (f) depodding efficiency; (g) depodded kernel; (h) undepodded kernel; (i) small broken kernel; (j) big broken kernel.

**Figure 10:**
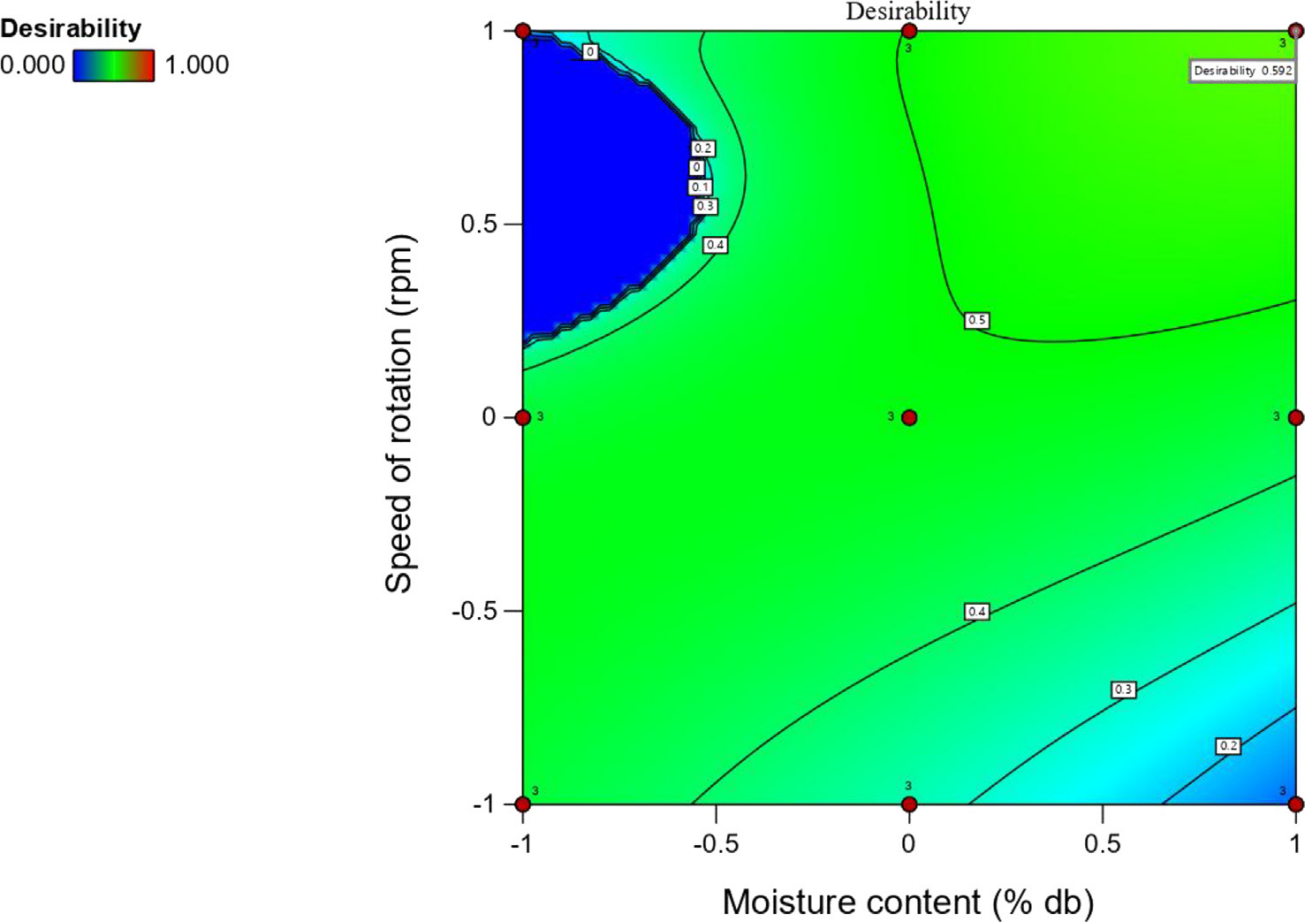
Desirability response surface plot of the performance efficiency of the developed moringa depodding machine.

The experimental and predicted values were in reasonable agreement for all the performance efficiencies evaluated at a desirability value of 0.62 (Figures 9a–j and 10). In the various range of moisture content (8.20–10.10% wet basis) and speed of rotation (365–584 rpm), in which the goal was to maximize the TP, ETP, DC, CW, DE, and minimize the LR, UK, SBK, and BBK. The optimal values predicted were TP (113.73 kg/h), ETP (109.45 kg/h), LR (0.85 man-hour required/Kg), DC (96.15 %), CW (0.96), DE (93.93 %), UK (0.02), SBK (10.64 %), and BBK (1.24 %) at moisture content of 10.10 % wet basis and speed of 564 rpm. At these optimal condition, the experimental values for TP, ETP, LR, DC, CW, DE, UK, SBK, and BBK were 112.41 kg/hr, 109.36 kg/h, 0.87 man-hour required/kg, 95.87 %, 0.98, 93.54 %, 0.03, 10.56%, and 1.23% respectively. The variation between the predicted and experimental values was non-significant suggesting that the models adopted in predicting the performance efficiencies of the developed depodding machine were reliable.

## 4 Conclusion

In this study, the effects of processing factors on the performance of the designed and fabricated moringa depodding machine using a response surface approach were evaluated. The response surface analysis revealed that the speed of rotation and crop moisture content had a significant effect on the various performance efficiencies (TP, ETP, LR, DC, CW, DE, UK, SBK, and BBK). The speed of rotation was found to have the greatest effect on the responses as compared to the moisture content within the experiment conducted. The effect of moisture content and speed of rotation were quadratic for TP, ETP, LR, DC, DE, UK, SBK, BBK, but was 2FI for CW. From the optimization study, the optimal values for the performance of the moringa depodding machine were recorded at the moisture content (10.10% wet basis) and speed of rotation (564 rpm). The predicted values for TP, ETP, LR, DC, CW, DE, UK, SBK, and BBK were 113.73 kg/h, 109.45 kg/h, 0.85 man-hour required/Kg, 96.15%, 0.96, 93.93%, 0.02, 10.64%, and 1.24% respectively. The predicted values were in a reasonable agreement with the experimented values with very little deviation for all responses considered. The empirical models derived for the TP, ETP, LR, DC, CW, DE, UK, SBK, and BBK was considered to sufficiently relate the observations.

## Declarations

### Author contribution statement

Clement Adekunle Komolafe & Clinton Emeka Okonkwo: Conceived and designed the experiments; Performed the experiments; Analyzed and interpreted the data; Wrote the paper.

Peter Pelumi Ikubanni: Conceived and designed the experiments; Wrote the paper.

Faith Olusola Ajao, Adewumi Samuel Alake & Tajudeen M. Adeniyi Olayanju: Performed the experiments; Wrote the paper.

### Funding statement

This research did not receive any specific grant from funding agencies in the public, commercial, or not-for-profit sectors.

### Competing interest statement

The authors declare no conflict of interest.

### Additional information

No additional information is available for this paper.
